# Report of the Committee on Diseases of the Eye

**Published:** 1866-07

**Authors:** E. L. Holmes

**Affiliations:** Chicago


*Report of the Committee on Diseases of the Eye.* By E. L. HOLMES, M. D., of Chicago.

(Read before the Illinois State Medical Society, June 6th, 1866.)

MR. PRESIDENT AND GENTLEMEN,—As your Committee on Diseases of the Eye, I would most respectfully submit the following Report:

It is to be regretted that other members of the society have not sent to your committee contributions upon some subject of Ophthalmology.

The experience of many of our members is so extensive in the treatment of many of the diseases of the eye, that much valuable knowledge regarding these diseases could be placed on record.

As a matter of great practical interest as well as importance, I venture to repeat what I have already brought to your notice at previous meetings in reference to the subject of conjunctivitis. It has been my intention to make this disease and its sequelæ the subject of a special paper for the consideration of this society. Although I have already promised such a paper, I may here state that I hope to present it at no distant time.

With increasing experience in the treatment of these diseases in patients from every portion of this State and the Northwest generally, I am the more convinced there are few affections so common and important and at the same time so neglected as these. Conjunctivitis is often so trifling, that it yields readily to the simplest treatment; often a most serious affection, which requires the utmost care and skill of the practitioner, and occasionally, it must be admitted, baffles the best resources of the oculist, however experienced, ruining the sense of sight, or leaving the eye much impaired in usefulness. Conjunctivitis is no exception to the rule that inflammation of any organ, however trifling it may at first appear, may resist the most skilful treatment, and yet there is scarcely a serious disease which we can so readily control by remedies, early and judiciously applied, as this.

Skill in the treatment of this disease depends principally upon the manner in which remedies are applied locally—upon the strength and frequency of the applications, more, perhaps, than upon the selection of the agent.

Internal remedies, although sometimes of very great importance, are often of secondary value—less valuable, we believe, than attention to quantity and quality of food and to protection of the eyes from all kinds of irritation.

There is scarcely anything worthy of particular notice, as indicating progress in the application either of new or of old remedies in this disease.

Chemical science multiplies almost without limit the number of agents which may be used with more or less favorable results. Experience shows, however, I think, that with quite rare exceptions, no advantage is gained by increasing the number of astringent salts used in the local treatment of conjunctivitis. Nothing has as yet superseded the old remedies. Other salts are valuable in such cases as resist all ordinary means.

The permanganate of potash is worthy of mention as an antiseptic in purulent conjunctivitis. It can be used in a solution of ten to twenty grains to the ounce of distilled water, dropped freely upon the conjunctiva at intervals between the applications of other remedies. There is reason to believe that the purulent matter, confined between the cornea and the folds of œdematous conjunctiva, which are so often observed in this disease, promotes ulceration of the cornea by its septic qualities.

The permanganate of potash is known to counteract the effects of this purulent matter. From the apparently good effects of this agent in a few cases, I am disposed to urge its use.

In the treatment of the diseases of the cornea there has been comparatively little advance during the past year. The subject of paracentesis of the cornea in cases of ulceration, hypopyum, and chronic inflammation, has attracted considerable attention among ophthalmologists. This little operation is especially indicated in deep ulcerations and abscesses of the cornea, and in cases of great pain with tendency to increased intraocular pressure.

The connection between the choroid and sub-conjunctival membrane is so intimate through vessels perforating the sclerotic, that there is often in conjunctivitis serious complications with the choroid and ciliary processes, which not only derange the nervous supply, but also increase the tension of the intra-ocular fluids. Paracentesis of the cornea removes this tension and seems to aid in restoring the nervous and vascular supply to their normal condition.

Since the ordinary puncture through the cornea soon closes, thus rendering its effects of short duration, an incision into the periphery of the anterior chamber with a cataract knife, with its edge directed backward, as proposed by Hancock, has been found in many cases a valuable means of relieving pain and overcoming tension of the globe. This incision does not close so readily as the ordinary minute opening made in the cornea by paracentesis, but allows the fluids to escape slowly for many hours.

Whether the good effects of this operation depend upon the form of opening into the anterior chamber and the consequent prolonged evacuation of the aqueous humor, or also upon the division of the ciliary muscle, is a matter of some doubt. The fact that in certain painful central ulcers of the cornea, the pain becomes less, as soon as the ulcer penetrates the cornea, either spontaneously or by puncture; and that the incision through the sclerotic in Hancock's operation seems most beneficial when it reaches the periphery of the anterior chamber and discharges the aqueous humor, would seem to indicate that in many cases at least the good results are independent of section of the ciliary muscle. Although a most valuable therapeutical agent in some forms of disease, it will still often fail to accomplish all that is desirable.

In reference to diseases of the iris, there is scarcely anything new to excite our interest. There is a tendency, to a greater or less extent, among oculists to seek remedies for the treatment of iritis without the use of mercury. Iodide of potash, turpentine, muriate of ammonia, have been used with good effects. Undoubtedly the most important agent is belladonna, which, in the form of a strong solution of neutral sulphate of atropine, should be instilled between the lids very frequently and from the very commencement of the attack. The failure to make an early diagnosis, and the neglect to bring the iris under the immediate influence of atropine before adhesions have taken place, are the chief causes why so many cases of iritis terminate with vision more or less seriously impaired.

Further experience is required to demonstrate the absolute value of other remedies as compared with calomel. I am confident, if members will employ atropine early and freely, they would find less necessity for using large doses of calomel.

If in any case treatment has been neglected, either on the part of the patient or physician, and the pupil becomes contracted and the iris adherent to the capsule of the lens, it should be always borne in mind that the subsequent danger from relapses is in proportion to the extent of the adhesions. Each relapse tends to increase the extent of the adhesions till, finally, the whole pupillary edge of the iris is attached to the crystalline lens. The aqueous humor collects between the lens and iris, forcing the latter forward towards the cornea. This condition cannot long continue without producing choroiditis and total loss of vision. The early removal of a portion of the iris, in cases of extensive adhesions, is not only almost certain to prevent relapses, but in cases, where the whole pupillary edge is adherent, will also restore the eye to sight.

I believe this important subject is overlooked by many practitioners, for the number of cases in which vision evidently might have been saved, but was lost from neglect of this operation, has not been small in my own experience.

In reference to the subject of cataract, there is much more to be said than the limits of this report can admit.

There is a marked tendency among the most distinguished oculists of the world to resort to the operation of extraction in nearly every form of cataract, whether hard or soft, except, perhaps, in infancy.

The removal of a portion of the iris, as in the operation for artificial pupil, seems to meet with almost universal approval among all oculists. This enables the whole cataract, nucleus and cortical substance to pass more readily through the iris with less danger of bruising it.

The more frequent use of atropine before and after the operation, for the sake of paralyzing the iris and ciliary muscle, is recommended, and I believe with much reason, by Græfe.

For a description of these modifications in the operation for extraction of cataract, I would refer to the extended articles of Critchett and Bowman in English, and of Græfe in German.

The use of the ophthalmoscope has become of more importance not only in the diagnosis of diseases of the intraocular structures but also for the investigation of diseases of certain other organs.

The use of this instrument is of special advantage to those who wish to investigate disease of the brain and nervous system. The ease with which the observer can look upon the retina and end of the optic nerve, (papilla,) and the fact that these nervous structures are so directly connected with the brain, not only by their nervous structure but also by their vascular supply, should awaken interest in all who study nervous disease. Several important monographs have been lately written upon the ophthalmoscope as a means of diagnosis of the affections of the brain.

The investigations which have thus far been made have failed to establish, except in a few diseases, such uniformity of appearances in the eye as will enable us to decide what abnormal conditions are dependent upon diseases of the brain and which are simply local manifestations of disease in the retina and optic nerve. Undoubtedly further research will extend our knowledge of this subject.

It is worthy of consideration that the use of the ophthalmoscope should no longer be confined to the specialist. The manner in which it is to be used is now so clearly explained; the plates illustrating the normal as well as the abnormal appearances in the choroid, retina, optic papilla and their vessels are now so numerous and accurate, that it requires comparatively little practice with the instrument to acquire reasonable skill in its use.

Ophthalmoscopic appearances in the choroid or retina can be readily understood by comparison with the plates in several well known works.

It cannot but be a matter of interest to this society to be informed that the clinical advantages for those wishing to pursue the study of diseases of the eye are, year by year, increasing in the city of Chicago. In the Cook County Hospital, recently opened for the treatment of the poor of the county generally, there has been opened a special ward for the treatment of diseases of the eye and ear. This portion of the hospital has been placed under the charge of Dr. J. S. Hildreth, and will be available to all students interested in ophthalmology.

The Chicago Charitable Eye and Ear Infirmary, now just entering upon the ninth year of its existence, is still in a most prosperous condition. Its capacity and accommodations have been recently much enlarged. Two hundred and sixteen patients have received the benefits of this charity during the past year. Ample facilities have been given the student for practical observation in the diagnosis of diseases of the eye with their medical and surgical treatment. During the war, there were treated at the Infirmary a large number of soldiers, soldiers' wives and children.

The Northwestern Sanitary Commission for some months paid the board of soldiers affected with diseases of the eye while under treatment at the Infirmary. There is still a small sum of money at the disposal of the Infirmary for the use of such discharged soldiers with diseases of the eye as may wish to obtain treatment.

It is most desirable that the friends of the Infirmary should not only enable it to bestow treatment and medicines gratuitously, as they now do, but also to support the patients while in its wards. It is to be hoped the benevolent people of this State will contribute as large a fund for this Infirmary as was established in Massachusetts and New York for the infirmaries of those States.

The Infirmary is open to members of the profession at all times for inspection.

I trust members of this society will not deem of little importance the general subject of specialties, and particularly the claims of ophthalmology as a specialty.

The fact that several of our public institutions for medical instruction have appointed special lecturers for the department of ophthalmology, is proof that the subject is of no small importance in their estimation. The fact that you, for several years, have appointed a special committee on oph thalmology, is sufficient to indicate how important you consider this subject.

Your committee is among those who believe most earnestly in the great good which can be accomplished by special attention directed to particular classes of diseases.

The science of medicine is too broad for one mind to cultivate with success its whole domain. While well directed efforts in the investigation of special subjects and in the treatment of diseases of special organs cannot fail to secure a degree of skill in diagnosis and treatment superior to that of general practitioners, it must not be forgotten that no one can become a reliable specialist without careful study of all the principles of medicine and without quite extensive experience in the diagnosis and treatment of disease of other organs.

It is often said by practitioners of experience that no physician should select his specialty till he had been at active practice several years. It is undoubtedly a fact that a student, who devotes four years to the careful study of medicine previous to taking his degree of doctor, and subsequently spends five years in study and practice, has not gained more important knowledge than is required to pursue with the best advantage some important specialty.

Ophthalmology we believe should, in a measure, be an exception to this rule. The science of this specialty is now not only so extensive, that a careful study of its theory and practice, with clinical experience sufficient for accurate diagnosis, requires a long period; but the operations are of such a nature, that they demand more than ordinary practice upon both the dead and living subject to secure perfection, or even tolerable skill.

It is not necessary to dwell upon the fact, that the recognition of abnormal changes in so delicate tissues as those of the eye requires long training of the eye on the part of the observer, and that many of the operations require a delicacy of manipulation which can only be acquired by long and patient practice.

There is scarcely an argument in favor of specialties more forcible than the fact, that nearly all the great original works in the various departments of our science have been written by specialists, or compiled from the fruits of their labors. Nearly all the great discoveries have been made by men who have devoted their energies to one special field of labor.

The science of ophthalmology affords one of the most interesting fields of study in the whole range of medicine. We can scarcely conceive of anything more delightful than the satisfaction of having restored the blind to sight. The good results of medical and surgical treatment are not more apparent in any branch of practice than in the management of diseases of the eye. The treatment of iritis, the operation for cataract and artificial pupil are specially worthy of high praise as wonders of science. While the efforts of the oculist are so often crowned with wonderful results; while the oculist himself finds so very much to encourage him and give him the satisfaction of having accomplished a great good, it must not be forgotten that his sense of responsibility and his anxieties are also very great. His patience is severely taxed in his efforts to relieve some of the more common but obstinate diseases. Patients often imagine that the oculist, if he is really well educated in his department, should in all cases prevent the loss of sight, and restore a diseased eye to health. Unfavorable results in a case of disease of the eye, often bring more condemnation upon the oculist, than even death in ordinary practice upon the general physician. In the one case the reproach is more lasting, for the patient is a living witness of the failure; in the other, the grave covers the proof of ill success, and all is soon forgotten; for the loss of an eye the medical attendant alone is blamed; the death of a patient is regarded as a dispensation of an all-wise Providence.

The true field of labor for the oculist is in a large city or in a densely settled country. It should not be the sole object of his ambition to accumulate wealth or secure a reputation by the skill he has attained, but also to act as a teacher. We do not wish to surround any of the operations upon the eye, even the most delicate, with mystery. It is true, however, that the most important of them do require much practice in their perfect performance and much judgment in the subsequent care and treatment of the patients. A thinly settled community cannot afford the specialist a sufficient number of cases to secure the requisite skill in the management of these important diseases.

Hence, as I have said, large cities are the appropriate field of labor for the oculist. While, in such cities, with the oculist near him, the general practitioner may neglect, if he chooses, particular attention to diseases of the eye without detriment to his patients, it is one of the special duties of the general practitioner in the country to make the ordinary diseases of the eye the subject of careful study, not only theoretically but practically.

This is an absolute demand which any community in the country can rightfully make upon the physician who seeks to practice medicine in it, for these diseases are common, and upon their family physicians alone must patients depend for advice and aid.

It is a remarkable fact that the common diseases of an organ so important as the eye should be neglected by so many practitioners of the West.

It would be difficult to estimate the number of people who become blind from incompetent medical advice and treatment in the early stages of inflammation of the different tissues of the eye.

There seems to be but one explanation of this neglect. In our medical colleges the subject has either been almost wholly ignored or received so little attention, that the graduate on commencing practice, finds it almost impossible either to make a diagnosis or advise a suitable course of treatment. Usually, he neglects through life what he has neglected in his preparatory studies.

In Europe, the most celebrated oculists are recognized as worthy of the high position they hold in the estimation not only of the public but of the medical profession.

In this country we have already many ophthalmologists who do honor to their specialty as well as the profession generally.

It is due to the high minded specialists of this country, that the profession discriminate between those who deserve full respect for their honor and faithfulness, and those who bring discredit upon their specialty by disregard for all that our profession holds sacred as it were; who perform the acts of charlatans, or who are eager to pursue as near as possible the course of dishonorable men, and yet pretend to the strictest obedience to the spirit of the code of ethics—the highest written law known to our profession. It is the course pursued by one class of specialists which has brought dishonor upon the whole system of specialties.

It is to be regretted that there is any tendency to misunderstanding and warm discussion between the conservative portion of our profession and the partizans of specialties. We hope no precipitate action will be taken on either side.

The differences of opinion are evidently more imaginary than real. It is doubtful whether any one, however conservative he may be, would object to the most devoted pursuit of a specialty, provided the medical education of those who thus devote themselves is such as fully fits them in all respects for its practice.

It is doubtful, on the other hand, whether any specialist of good reputation in our country would advocate the pursuit of a specialty in a manner which would tend to narrow the views of the practitioner.

It is probably a well established fact that the young man who spends four years in the study of language and the sciences previous to the commencement of his three years course of medical studies will be better prepared, all things considered, to perform the active duties of his profession, than one who devotes the whole seven years to the medical sciences alone.

So also the student of medicine should devote himself for years most earnestly to the study and practice of his profession before devoting his best energies to a single specialty. I have already expressed my opinion in regard to the limits within which the oculist might with propriety, from the very beginning, give his attention to his specialty.

Any system of education is to be strenuously deprecated, which would permit a student to select for study those portions alone of anatomy, physiology, materia medica and surgery, which he might consider as bearing directly upon his specialty.

I would consider the establishment of special infirmaries for the treatment of diseases of the eye and ear as a matter of great importance. Such patients, being usually in comparatively good general health, are in far better circumstances in an infirmary than they can be under the same roof with patients suffering from typhoid fever, erysipelas, suppurating wounds and other diseases, which render all our best means of ventilation in general hospitals, to a certain extent, ineffectual. Such infirmaries should be open for all students who wish to extend their knowledge of diseases of the eye or ear.

I take pleasure in stating that an American Ophthalmological Society has been formed, and is now in successful operation, which is composed of members, many of whom are well known throughout the country, as well as in Europe. I believe this society will accomplish much for science, and will tend to inspire the whole profession with the desire to place ophthalmology in the position it should hold, and to lead all students of medicine to make it the subject of careful study.

As a most earnest advocate of specialties in large cities and densely populated communities, I trust no course will ever be directly or indirectly recommended which will tend to lead the specialist to pursue a superficial course of medicine, or to increase the danger that the general practitioner in the country will neglect ophthalmology, on the ground, that it is the proper sphere of the specialist alone. These results must inevitably follow an injudicious subdivision of professional labor.

